# Attachment Style of Volunteer Counselors in Telephone Emergency Services Predicts Counseling Process

**DOI:** 10.3389/fpsyg.2019.01936

**Published:** 2019-08-30

**Authors:** Ulrike Dinger, Simone Jennissen, Isabelle Rek

**Affiliations:** Department of General Internal Medicine and Psychosomatics, University Hospital Heidelberg, Heidelberg, Germany

**Keywords:** attachment, helpline, telephone emergency service, paraprofessional volunteers, counseling, therapeutic alliance

## Abstract

Telephone emergency services (TES) provide emotional support and aim to prevent suicide. The current study examines a potential change of volunteer counselors’ attachment characteristics during TES training and investigates the predictive influence of counselor attachment for their competence and working alliance with callers. We recruited 261 volunteers enrolled in training for paraprofessional counseling in the German Telephone Emergency Service (*TelefonSeelsorge*). Participants were assessed three times during their training (mean training duration 13.3 months) and responded to questionnaires on adult attachment (Experience in Close Relationships-Revised) and their counseling competence (adapted Development of Psychotherapists Common Core Questionnaire). In addition, they indicated the quality of the working alliance (adapted Working Alliance Inventory – Short, Revised) with their client callers upon training completion. Results showed that attachment anxiety, but not attachment avoidance, significantly decreased during training. Lower attachment avoidance predicted better working alliances with callers as well as better general skillfulness. Implications for the training of volunteer telephone counselors are discussed.

## Introduction

Telephone emergency services (TES) are an important component of psychosocial health care and suicide prevention strategies across different healthcare systems. Their offer is readily available, usually free of charge and confidential. The International Federation of Telephone Emergency Services (IFOTES) comprises 31 national federations and associations of 23 countries, including the German TES *TelefonSeelsorge*. The TelefonSeelsorge reported a total of 8.7 million calls made in Germany in 2016. About 50% of the callers repeatedly contacted the TES and around 30% of the callers reported to suffer from a diagnosed mental disorder. The most frequent themes callers talked about were depressive mood, anxiety, physical health problems, and interpersonal problems in families and romantic relationships (TelefonSeelsorge, 2016). Comparable contents have been reported for TES calls in the UK (Coveney et al., 2012), North America (Mishara et al., 2007; Ingram et al., 2008), and Australia (Burgess et al., 2008). These topics are highly similar to those discussed in face-to-face counseling and psychotherapy. Compared to regular face-to-face counseling settings, less is known about the counseling process and influential factors in the TES setting.

In psychotherapy and counseling, the person of the therapist is an important factor for the success of the therapy. Therapists account for approximately 5% of their patients’ outcome variance and further differ in the quality of the therapeutic alliance with their patients (Baldwin and Imel, 2013). To date, it is not fully understood which therapist characteristics are responsible for more or less successful treatments. A recent systematic review on therapists in psychodynamic therapy concluded that the results on therapists’ general personality characteristics (i.e. Big-5 traits) are heterogeneous, but that therapists’ typical interpersonal patterns consistently affect treatment outcome across primary studies (Lingiardi et al., 2018).

Compared to the general population, psychotherapist trainees are characterized by lower attachment avoidance and more affiliative interpersonal values (Rek et al., 2018). A previous longitudinal study showed that therapists’ introject affiliation may increase during therapists’ training and personal therapy (Taubner et al., 2013). Therapists’ attachment characteristics appear to be relevant predictors for their professional relationship skills and thereby influence the therapeutic process. Although further research in this domain is clearly needed, two recent systematic reviews concluded that the therapist’s attachment style contributes significantly to alliance and patient outcome (Degnan et al., 2016; Steel et al., 2018). Some studies showed that greater attachment security of the therapist is beneficial, especially when working with severely impaired patients (e.g., Bruck et al., 2006; Schauenburg et al., 2010). Among the specific insecure strategies, therapists’ attachment preoccupation appears to be most problematic (e.g., Dinger et al., 2009; Petrowski et al., 2011).

Compared to face-to-face counseling and psychotherapy, TES counselors remain understudied and research on the association between TES counselor variables and the counseling process is scarce. One major difference to other psychosocial treatment offers is that TES are typically provided by paraprofessional volunteer counselors. This is due to the fact that TES counseling is often free of charge and available around the clock (24/7). Previous studies showed that adequate training for paraprofessional counselors is important, because the quality of their counseling increased with specific counseling training of sufficient duration. Compared to lay individuals, paraprofessional volunteers with adequate training reacted more flexible in an online support group, used a wider range of interventions in response to suicidal messages, and utilized more cognitive-focused and emotion-focused counseling strategies (Gilat et al., 2012). Skills training for interventions against suicide within the U.S. National Suicide Prevention Lifeline network resulted in fewer symptoms of suicidal callers (Gould et al., 2013). These findings support the necessity of sufficient counseling training for volunteer counselors in the TES context. Training in the German TelefonSeelsorge includes theoretical classes, practical counseling exercises (e.g., role-play), supervision, and personal counseling in a group context. The focus of the personal counseling groups is to increase the volunteers’ self-understanding of interpersonal dynamics. Depending on the local institution, the duration of training varies between 6 and 24 months (Rek and Dinger, 2015).

Regarding the personality characteristics of volunteer telephone counselors, a previous study compared the Big Five personality traits, interpersonal empathy, and mental health difficulties of 54 volunteers of a student delivered telephone helpline to the personality characteristics of 52 non-volunteer students. Student telephone counselors indicated more agreeableness and more empathy (perspective taking and empathetic concern), but did not differ from non-volunteer students regarding their self-reported mental health (Paterson et al., 2009). Within the German TES TelefonSeelsorge, Rek and Dinger (2016) compared interpersonal motives of 261 volunteer trainees at the beginning of their paraprofessional training to a matched non-clinical reference group from a representative survey. On average, the volunteer counselors reported significantly higher scores on harmonious and helpful influential interpersonal motives. A cluster analysis indicated three distinct trainee subgroups, which were characterized by mainly submissive-altruistic, helpful-influential, and friendly-harmonious interpersonal motives.

To our knowledge, there are no previous studies on the association of TES counselors’ interpersonal or attachment characteristics with the process and outcome of telephone counseling. The current longitudinal study aims to investigate the association between volunteer counselors’ attachment characteristics, their potential change during training, and their predictive value for counseling competence after training completion. Attachment characteristics are assessed *via* self-reported partner-based attachment anxiety and avoidance at three time points during training for TES counseling. Counseling competence is operationalized *via* the average working alliance with callers and counselors’ self-perceived counseling skills at the end of TES training. It is unclear whether volunteers’ attachment characteristics remain stable over the training period, or whether the personal counseling groups during training help to change attachment insecurities. Based on the findings that group psychotherapy can lead to positive changes in attachment security (Kirchmann et al., 2012; Maxwell et al., 2014) and that psychotherapist trainees in personal therapy previously reported an increase in introject affiliation (Taubner et al., 2014), we expect a reduction of attachment anxiety and avoidance during counseling training. We further aim to investigate the associations between attachment characteristics and counseling competence. Based on the previous findings with psychotherapists (Degnan et al., 2016; Steel et al., 2018), we expect that TES volunteers’ attachment characteristics predict their counseling competence. More specifically, we hypothesize that lower levels of attachment anxiety and avoidance are associated with better working alliances and more competent counseling skills. We also aim to examine whether observed changes in attachment anxiety and avoidance (i.e. their slope of change) during training predict counseling competence upon training completion.

## Materials and Methods

### Participants and Procedure

The participants were recruited for a prospective longitudinal study designed to assess the paraprofessional development of TES volunteer counselors during their training. Of 333 eligible volunteer counselors, 261 participants agreed to participate and completed the questionnaire at the beginning of TES training. Of these, 193 (73.9%) were women, their mean age was 52.21 years (SD = 10.21, range: 19–73). Their level of education was mixed and ranged from basic secondary schooling to university degrees. The majority of participants were currently employed in addition to their volunteer work for TES. Approximately two-thirds (65.2%) of the participants lived together with their spouse or romantic partner (see Table 1 for detailed sample characteristics).

**Table 1:** TES counselor trainee sample (*N* = 261).

	Mean	SD
Age (years)	52.21	10.21
	*N*	%
Gender female	193	73.9
**Education^1^**		
Basic secondary school	67	25.7
Advanced secondary school (“Abitur”)	76	29.1
College/university degree	117	44.8
**Employment**		
No work (e.g., students, retired)	53	20.3
Part-time	109	41.7
Full-time	75	28.7
Other (e.g., military service)	24	9.3

1 *The German school system includes basic secondary schools (until 10th grade) and advanced secondary schools (until 13th grade) with entrance qualification for university*.

Participants responded to questionnaires at three measurement points: (1) at the beginning of training, (2) after 6 months of training, and (3) after the completion of counseling training. The mean training duration was 13.3 months (SD = 3.64, Range: (7–24). For recruitment, the professional directors of TES institutions were informed about the study at the annual national meeting of German TES directors. In case of agreement to participate and the start of a new training group during the study period, local TES directors and training instructors informed and recruited volunteer trainees at their respective site. Volunteer trainees were also invited to contact the study authors for any questions they might have. In case of written informed consent, volunteer trainees received the study questionnaires from their local TES directors at their first training session and were asked to return the questionnaires at the next session. Questionnaire items were presented in the standard order at each assessment (i.e., no randomized sequence). The same procedure was used after 6 months of training and after completion of the training. In addition, participants filled out the working alliance inventory (WAI-SR) for each one of their first five independent TES calls after the completion of training, resulting in five alliance assessments with different callers per counselor. Study participation for volunteer trainees was voluntary and without financial compensation. Questionnaires were pseudonymized and sent to the study authors with any personal contact information removed.

Out of the 261 participants, 226 completed the questionnaires after 6 months (87% of the initial sample) and 180 participants returned the questionnaires (69% of the initial sample) after training completion. Out of the 81 study dropouts, 32 participants ended the training prematurely (22 within the first 6 months) and 24 completed their training without further study participation (9 decided to drop out of the study within the first 6 months). The remaining participants were either still in training at the time of the data analysis (11 participants) or no information was available on them (14 participants).

### Measures

#### Attachment

The ECR-R (Fraley et al., 2000) measures self-reported adult attachment in romantic relationships. It is the most widely used attachment questionnaire to date and therefore allows a comparison of the current study findings with other samples. The 36 items load on two factors: attachment anxiety indicates fear of abandonment and rejection (item example: “I’m afraid that I will lose my partner’s love.”), while attachment avoidance indicates discomfort with interpersonal closeness and intimacy (item example: “I don’t feel comfortable opening up to romantic partners.”, Brennan et al., 1998; Fraley et al., 2000). Respondents are asked to indicate their agreement on a 7-point Likert scale ranging from 1 (strongly disagree) to 7 (strongly agree). The German translation of the ECR-R (Ehrenthal et al., 2009) shows excellent psychometric properties. In the current study, Cronbach’s alpha on the first assessment was *α* = 0.91 for attachment anxiety and *α* = 0.90 for attachment avoidance.

#### Counseling Competence

The German version of the Technical Expertise and Basic Relational Skills of Development of the Psychotherapist Common Core Questionnaire (DPCCQ; Orlinsky et al., 1999; Orlinsky and Rønnestad, 2005) was adapted for the current study in order to assess telephone counselors’ self-rated technical expertise and relational skills. The SPR Collaborative Research Network translated the original DPCCQ into German (Orlinsky et al., 1999). Twelve items were adapted for the TES context [Technical Expertise, item example: “How well do you understand what whappens moment-by-moment during *helpline counseling calls* (replaced ‘therapy sessions’)?”; Basic Relational Skills, item example: “How effective are you in communicating your understanding and concern to your *callers* (replaced ‘patients’)?”; and Advanced Relational Skills, item example: “How well are you able to detect and deal with your *callers’* (replaced ‘patients’) emotional reactions to you?”], which measures the degree of current skills. The items were answered by the counselor trainees on a 6-point Likert scale from 0 (not at all) to 5 (very) at the end of the TES training. The mean of the three subscales, Technical Expertise, Basic Relational Skills, and Advanced Relational Skills, were used as indicators of self-reported current skillfulness. In the current study, Cronbach’s alpha for the adapted current skillfulness was *α* = 0.88.

#### Working Alliance

The Working Alliance Inventory-Short Revised (WAI-SR; Hatcher and Gillaspy, 2006) is a 12-item short version of the original WAI (Horvath and Greenberg, 1989) with three subscales based on Bordin’s (1979) working alliance theory: goals (assessing agreement about overall treatment goals), tasks (assessing agreement on the appropriate tasks to achieve the goals), and bond (assessing the trust and rapport between therapist and client). In the current study, the German therapist version of the WAI-SR was used (WAI-SR; Wilmers et al., 2008) to assess the alliance with callers during counselors’ first five independent calls after training completion. When appropriate, the items were adapted for the TES setting [Tasks, item example: “*The caller* (replaced ‘patient’) and I agree about the steps to be taken to improve his/her situation.”; Bond, item example: “*The caller* (replaced ‘patient’) and I respect each other.”); but remained unchanged in other cases (Goals, item example: “We are working towards mutually agreed upon goals.”)]. The counselor trainees rated each item on a 7-point scale ranging from 1 (never) to 7 (always). In the present study, Cronbach’s alpha for the first alliance assessment was *α* = 0.94 for the adapted WAI-SR-T mean score.

### Data Analytic Strategy

Deviations from normality were deemed negligible based on sample size and visual inspections of histograms. There were no multivariate outliers in the data according to Mahalanobis’ distance (*α* = 0.001). Hypotheses on the change of attachment characteristics during training were examined using repeated-measures analysis of variance (ANOVA). The assumption of sphericity was assessed with Mauchly’s test and Greenhouse Geisser corrections were applied if violations were detected. Bonferroni-corrected *post-hoc* tests were used to identify which specific means differed. Effect sizes for ANOVAs were calculated as $\eta_{p}^{2}$, with values of 0.02, 0.13, and 0.26 representing small, medium, and large effects, respectively (Cohen, 1988). These statistical analyses were performed using SPSS version 21.

Since we were interested in testing whether the levels, as well as the changes in attachment characteristics predict counseling competence, we employed a growth curve modeling approach to test these hypotheses simultaneously. Growth curve models allow for an assessment of the rate of change (level) as well as the shape of change (slope) that characterizes a sample of individuals (Little, 2013). These models estimate between-person differences in within-person change (Curran et al., 2010). To estimate reliable parameters, variables therefore need to show between- as well as within-person variability. Level and slope parameters can then be used as predictors of outcome variables. Since counseling competence was operationalized *via* two different variables, namely average working alliance (across counselors’ first five independent telephone calls) as well as general self-perceived skillfulness, we predicted these outcome variables in separate models. Growth curve analyses were performed with the R package *lavaan* version 0.5-23 (Rosseel, 2012). Slopes were modeled as linear trends. Missing data were handled with full-information maximum likelihood estimation. Model fit was evaluated with the *χ*^2^ fit statistic, the Comparative Fit Index (CFI), the Standardized Root Mean Square Residual (SRMR), and the Root Mean Square Error of Approximation (RMSEA). Nonsignificant *χ*^2^ values indicate model fit, while CFI values ≥ 0.95, SRMR values ≤ 0.08, and RMSEA values ≤ 0.06 suggest good fit (Hu and Bentler, 1999; Kline, 2011).

## Results

### Change of Attachment Anxiety and Attachment Avoidance During Training

Descriptive statistics for attachment anxiety and avoidance are presented in Table 2. There was a significant decrease in attachment anxiety across time (*F*_(*N* = 162, df = 1.89)_ = 6.02, *p* = 0.003, $\eta_{p}^{2}$ = 0.036). Mauchly’s test indicated that the assumption of sphericity had been violated (*χ*^2^(2) = 9.34, *p* = 0.009). We therefore report Greenhouse-Geisser corrected tests. *Post-hoc* test revealed a significant reduction between time one and time two (*p* = 0.026), as well as between time one and time three (*p* = 0.006). Attachment avoidance did not significantly decrease across measurement points (*F*_(*N* = 162, df = 2)_ = 2.12, *p* = 0.12, $\eta_{p}^{2}$ = 0.013).

**Table 2:** Attachment, counseling skills, and working alliance in TES counselor trainees.

	T1		T2		T3		Test of difference	
	*N*	*M* (SD)	*N*	*M* (SD)	*N*	*M* (SD)	*F*	*η*^2^
**Attachment**								
Anxiety	249	2.45 (1.02)	217	2.29 (0.86)	173	2.24 (0.95)	6.02^**^	0.036
Avoidance	248	2.24 (0.94)	217	2.26 (0.90)	173	2.15 (0.86)	2.12	0.013
**Counseling skills**								
Total	–	–	–	–	174	3.61 (0.49)	–	–
**Working alliance**								
Total	–	–	–	–	151	3.35 (0.50)	–	–

*T1 = at beginning of training; T2 = after 6 months of training; T3 = after completion of training. η^2^ = measure of effect size in analysis of variance. A small effect size is considered to be 0.010–0.058 (Kirk, 1996). Kirk’s criteria are for omega-squared; however, these criteria may be appropriately applied to interpreting partial eta-squared which is a similar measure of strength of association*.

** *p < 0.01*.

### Prediction of Counseling Competence by Attachment Anxiety and Attachment Avoidance

**Figure 1:**
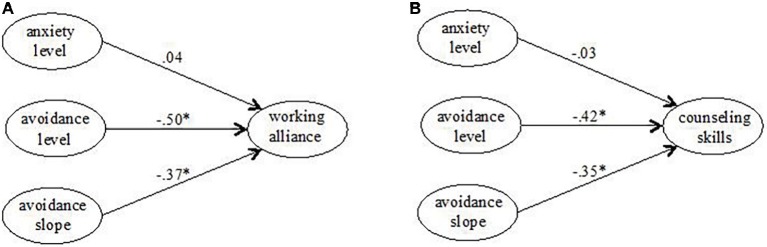
Growth curve models predicting working alliance with callers **(A)** and self-perceived counseling skills **(B)** from attachment anxiety and attachment avoidance. Path coefficients were standardized for model display. Significance levels refer to unstandardized parameter estimates. *p < 0.05.

We first tested whether or not the study variables were related to age or gender. Neither the attachment scales nor working alliance or current skillfulness were significantly associated with age or gender. However, a difference between men and women for current skillfulness approached significance level. Women tended to rate their current skillfulness slightly higher than men (Mean females 3.65, SD 0.46, mean males 3.48, SD 0.51, mean; *t*(166) = 1.97, *p* = 0.05). In addition to attachment anxiety and avoidance, Table 2 presents descriptive statistics for counseling competence and working alliance. Figure 1 depicts two growth curve prediction models for counseling competence operationalized as average working alliance with callers (Figure 1A) and counselors’ self-perceived counseling skills (Figure 1B). The model predicting average working alliance across counselors’ first five telephone calls from attachment anxiety and avoidance demonstrated good model fit (*χ*^2^_(*N* = 257, df = 43)_ = 75.14, *p* = 0.002; CFI = 0.96, RMSEA = 0.054, SRMR = 0.062). There was a significant negative association between both the levels (*β* = −0.50, *b* = −0.20, SE*_b_* = 0.08, *p* = 0.009) as well as the slope (*β* = −0.37, *b* = −0.42, SE*_b_* = 0.21, *p* = 0.042) of attachment avoidance and working alliance. This indicates that lower levels as well as a more significant decrease in attachment avoidance during training predict higher levels of working alliances with callers. Attachment anxiety did not emerge as a significant predictor. Since there was insufficient between-person variability in the slope of attachment anxiety, the association between the slope of attachment anxiety and working alliance was fixed to zero. This did not change the pattern of results.

The model predicting counselors’ self-perceived current skillfulness from attachment anxiety and avoidance yielded similar results. Overall, the model showed acceptable to good model fit (*χ*^2^_(*N* = 256, df = 24)_ = 74.72, *p* < 0.001; CFI = 0.95, RMSEA = 0.091, SRMR = 0.069). Again, there was a significant negative association between both the level (*β* = −0.42, *b* = −0.18, SE*_b_* = 0.06, *p* = 0.004) as well as the slope (*β* = −0.35, *b* = −0.44, SE*_b_* = 0.17, *p* = 0.009) of attachment avoidance and self-perceived counseling skills. Attachment anxiety did not emerge as a significant predictor. The slope of attachment anxiety was again omitted as a predictor due to its low between-person variability. Again, this did not change the pattern of results.

## Discussion

The current study examined the predictive influence of attachment anxiety and avoidance, as well as its potential change over time on their counseling competence and working alliance with callers.

### Change of Attachment Styles

Attachment anxiety, but not attachment avoidance, significantly decreased during training within the counselor sample. The empirical findings revealed that lower levels of attachment avoidance as well as a greater decrease in attachment avoidance during training predicted better working alliances with callers and higher skillfulness upon training completion. The interpretation of the observed change in attachment anxiety, but not avoidance, over time can be facilitated by a comparison of the level of attachment anxiety and avoidance with other samples. Compared to a large nonclinical German group of 1,006 individuals who answered the ECR-R during its psychometric evaluation (Ehrenthal et al., 2009), the telephone counselors were slightly less anxious, but similarly avoidant. However, this nonclinical comparison group was not representative for the general population. A recent study compared attachment characteristics of psychotherapist trainees in either CBT or psychodynamic psychotherapy training programs with an age- and gender-matched reference group from a representative population survey and found lower attachment anxiety, but no difference in avoidance among the psychotherapist trainees (Rek et al., 2018). A descriptive comparison of Rek et al.’s (2018) therapist sample with the current sample of TES counselor volunteers indicates similar attachment anxiety between both samples (*M*_TES_ = 2.45, SD = 1.02; *M*_Therapists_ = 2.46, SD = 1.12), but slightly higher scores on attachment avoidance among the TES volunteers (*M*_TES_ = 2.24, SD = 0.94; *M*_Therapists_ = 2.01, SD = 0.82). One explanation for the slightly higher scores on attachment avoidance in the TES trainee sample compared to the therapist sample might be that volunteering in helplines *via* telephone allows a greater interpersonal distance than psychotherapy with patients in a face-to-face contact. Consequently, some people might be attracted to volunteering in helplines, who feel a greater need to regulate the closeness of contact despite their wish to be helpful to others.

In addition to the descriptive differences to other samples, we were interested in the potential changes of attachment anxiety and avoidance. Because the German TES training involves the participation in a personal counseling group (Dinger and Rek, 2017), we expected that attachment insecurities might change during training. The review by Taylor et al. (2015) suggests that attachment security might improve during psychotherapy. More specifically, previous studies showed that attachment anxiety decreases in successful therapies, while the findings on attachment avoidance are less clear. Positive changes in attachment security and a reduction in both attachment-related anxiety and avoidance have been also identified after group therapy (Kirchmann et al., 2012; Marmarosh and Tasca, 2013; Maxwell et al., 2014). Although the TES volunteers in these studies were neither patients nor particularly insecure with regard to attachment anxiety at the beginning, they nevertheless experienced a reduction in attachment anxiety during their period of training. We currently attribute this change to the personal counseling group.

Although the TES counselors’ level of attachment avoidance was slightly higher than their degree of attachment anxiety, their average avoidance did not change over the course of training. Since this is in line with the mixed finding for change in attachment avoidance during psychotherapy, we suspect that attachment avoidance might be more difficult to change in counseling or therapy. This might be due to the fact that attachment avoidance often goes along with a more positive self-image and a suppression of painful, “needy” feelings. This might be a more functional interpersonal coping mechanism for the counselor trainees and therefore more difficult to give up than high levels of attachment anxiety, which is associated with rumination, self-blame, and internalizing behaviors (Morley and Moran, 2011; Malik et al., 2015; Schauenburg, 2016).

### Prediction of Counseling Competence

Regarding the descriptive statistics of the outcome variables at the end of training, the beginning volunteer TES counselors descriptively rated the experienced working alliance with callers substantially lower than a recently published therapist sample (31 experienced therapists with either CBT or IPT orientation) rated the working alliance with their face-to-face psychotherapy patients (Falkenström et al., 2016; *M*_TES_ = 3.35, SD = 0.50; *M*_Therapists_ = 5.19, SD = 0.91). On the other hand, the TES counselors descriptively rated their level of currently skillfulness similarly compared to the 4,923 practicing psychotherapists in the Collaborative Research Network sample from Orlinsky and Rønnestad (2005); (*M*_TES_ = 3.61, SD = 0.49; *M*_Therapists_ = 3.61, SD = 0.65). One explanation for these results might be that the volunteer counselors feel well prepared regarding their general counseling skills after TES training, but nevertheless experience it as difficult to establish a positive working alliance with callers *via* a single telephone conversation. This might be due to the open nature of the TES setting (e.g., open duration of calls) and the absence of a clear definition of goals in a singular helpline contact. This study was the first to administer an adapted version of the WAI-SR in the TES context. Further studies are needed to evaluate the psychometric properties and the suitability of the adapted WAI-SR in the TES context. In addition, there was a statistical trend for higher current skillfulness in female volunteers. Meta-analyses from psychotherapy research indicate negligible gender effects for counseling processes or outcome variables (Beutler et al., 2004). Although the gender effect here is not significant, it is possible that this is different in the TES setting. Female volunteers might feel more comfortable with counseling *via* the telephone, which would correspond to the fact that they generally spend more time talking on the phone than men (e.g., Smoreda and Licoppe, 2000).

The growth curve prediction models indicated that lower levels of attachment avoidance at the beginning of training as well as a greater decrease in attachment avoidance during training predicted better working alliances and better general skillfulness. It is important to note that on average, attachment avoidance did not change significantly during training. However, there was significant inter-individual variation, with some counselors decreasing and others increasing in attachment avoidance. Despite the non-significant average change in attachment avoidance during TES counselor training, the growth curve finding suggests that attachment avoidance is an important TES counselor characteristic. This is in line with previous findings from face-to-face counseling and psychotherapy research. In addition to other variables, Dunkle and Friedlander’s (1996) study showed that therapists’ comfort with closeness in interpersonal relationships was predictive for clients’ ratings of the bond component of the working alliance. The study by Mohr et al. (2005) reported that while counselors’ attachment was not predictive for clients’ evaluation of the session, their dismissive attachment was associated with more hostile countertransference as rated by supervisors. Romano et al. (2009) found an interaction effect of trainee therapist and client attachment. In their study, therapists with avoidant attachment used more directive interventions when clients were also high in attachment avoidance.

The current study in the TES setting differs from these previous studies. Because neither the telephone counselor nor the caller can rely on nonverbal information (e.g., facial expressions or gestures) during their telephone counseling, the counselor-caller interactions in the TES context may be more strongly influenced by both interaction partners’ interpersonal expectations. In addition, the telephone contacts are usually singular and may be interrupted spontaneously. Given the current findings, the paraprofessional TES training should focus on the potential negative effects of avoidance-related behavior (i.e. minimizing of distress, overemphasizing of independence, quick search for behavioral solutions). In addition, we consider it especially important for TES volunteer counselors to gain knowledge about their typical interpersonal “pitfalls” and to receive training on how to deal with these in their interaction with helpline callers.

### Strengths and Limitations

The current longitudinal study examined the association between volunteer counselors’ attachment characteristics, their potential change during training, and their ability to predict counseling competence after training completion in the TES context. The sample was adequately powered and the longitudinal design allowed the assessment of both between-person as well as within-person latent effects for attachment. We chose the current SEM modeling procedure over a 3-level hierarchical multilevel model, because the former allows for an estimation of latent within and between effects without measurement errors. At the same time, this procedure did not allow a statistical control of random site effects.

While both the age and gender distribution of the current sample (Blömeke, unpublished) as well as the training content and duration of their training (Rek and Dinger, 2015) is typical for TES volunteers in Germany, telephone helpline settings and training for volunteer counseling are highly diverse across countries and settings. It is currently unclear whether the findings can be generalized to other volunteer groups, e.g., to student helpline volunteers. The voluntary participation of study participants might have resulted in a positive selection bias. Because we did not assess the field of work or study (for students), it is not possible to control for a potential influence of this variable. It is possible that for example volunteers with a professional background in mental health have greater previous counseling experience and are more skilled compared to other volunteers. Another limitation of this study is the use of self-reports only, which might be influenced by social-cognitive biases and social desirability. In addition, the original versions on DPCCQ counseling skills and WAI-T working alliance were adapted here. This was done in order to compare the TES counseling process with previous findings from face-to-face counseling and psychotherapy research, but there was no previous study on their psychometric property and suitability in the TES counseling context. Furthermore, future studies should include longer term follow-up assessments after completion of training in order to examine the predictive importance of attachment characteristics and personal development during training for telephone emergency counseling.

## Conclusion

Attachment characteristics of volunteer telephone counselors are relevant for their competent counseling of helpline callers. Similar to the findings for psychotherapists (e.g., Degnan et al., 2016; Steel et al., 2018), we consider it important that TES counselor volunteers direct attention to the influence of their own attachment pattern in the telephone counseling process. A better self-understanding of their personal attachment styles can help counselors to raise awareness to those emotional responses to their callers that are influenced by their own personality. This might eventually facilitate better helping relationships with their callers. We recommend that training and supervision should focus specifically on the influence of counselors’ attachment avoidance on the helping alliance with callers.

## Ethics Statement

The study was approved by the ethics committee at the Medical Faculty, Heidelberg University (S-362/2014). All participants gave written informed consent following the Declaration of Helsinki.

## Author Contributions

UD designed the study, supervised the data collection and statistical analysis, and wrote a first draft of the manuscript. SJ performed the statistical analyses together with IR and critically revised the manuscript. IR contributed to the study design, coordinated the data collection, performed the statistical analyses together with SJ, and critically revised the manuscript. All authors approved the final version of the manuscript prior to submission.

### Conflict of Interest Statement

The authors declare that the research was conducted in the absence of any commercial or financial relationships that could be construed as a potential conflict of interest.

## References

[ref1] Baldwin S. A. Imel Z. E. (2013). “Therapist effects” in Bergin and Garfield’s handbook of psychotherapy and behavioral change. 6th Edn. ed. Lambert M. J. (New York: Wiley), 258–297.

[ref2] Beutler L. E. Malik M. Alimohamed S. Harwood T. M. Talebi H. Noble S. (2004). “Therapist variables” in Bergin and Garfield’s handbook of psychotherapy and behavior change. 5th Edn. ed. Lambert M. J. (New York: Wiley), 227–306.

[ref3] Bordin E. S. (1979). The generalizability of the psychoanalytic concept of the working alliance. Psychother. Theory Res. Pract. 16, 252–260. 10.1037/h0085885

[ref4] Brennan K. Clark C. Shaver P. (1998). “Self-report measurement of adult attachment: an integrative overview” in Attachment theory and close relationships. eds. Simpson J. Rholes W. (New York: The Guilford Press), 46–75.

[ref5] Bruck E. Winston A. Aderholt S. Muran J. C. (2006). Predictive validity of patient and therapist attachment and introject styles. Am. J. Psychother. 60, 393–406. 10.1176/appi.psychotherapy.2006.60.4.393, PMID: 17340948

[ref6] Burgess N. Christensen H. Leach L. S. Farrer L. Griffiths K. M. (2008). Mental health profile of callers to a telephone counselling service. J. Telemed. Telecare 14, 42–47. 10.1258/jtt.2007.070610, PMID: 18318929

[ref508] Cohen J. (1988). Statistical power analysis for the behavioral sciences. 2nd Edn. Hillsdale, NJ: Erlbaum.

[ref7] Coveney C. M. Pollock K. Armstrong S. Moore J. (2012). Callers’ experiences of contacting a national suicide prevention helpline: report of an online survey. Crisis 33, 313–324. 10.1027/0227-5910/a000151, PMID: 22759662 PMC3643796

[ref509] Curran P. J. Obeidat K. Losardo D. (2010). Twelve frequently asked questions about growth curve modeling. J. Cogn. Dev. 11, 121–136. 10.1080/15248371003699969 21743795 PMC3131138

[ref8] Degnan A. Seymour-Hyde A. Harris A. Berry K. (2016). The role of therapist attachment in alliance and outcome: a systematic literature review. Clin. Psychol. Psychother. 23, 47–65. 10.1002/cpp.1937, PMID: 25445258

[ref9] Dinger U. Rek I. (2017). Effekte der Seelsorgeausbildung Ehrenamtlicher - Ergebnisse eines empirischen Forschungsprojekts in der Telefonseelsorge. Pastoraltheologie 106, 469–498. 10.13109/path.2017.106.12.469

[ref10] Dinger U. Strack M. Sachsse T. Schauenburg H. (2009). Therapists’ attachment, patients’ interpersonal problems and alliance development over time in inpatient psychotherapy. Psychotherapy 46, 277–290. 10.1037/a0016913 22122718

[ref11] Dunkle J. H. Friedlander M. L. (1996). Contribution of therapist experience and personal characteristics to the working alliance. J. Couns. Psychol. 43, 456–460. 10.1037/0022-0167.43.4.456

[ref12] Ehrenthal J. C. Dinger U. Lamla A. Funken B. Schauenburg H. (2009). Evaluation der deutschsprachigen version des Bindungsfragebogens “experiences in close relationships – revised” (ECR-RD) [evaluation of the German version of the attachment questionnaire “experiences in close relationships-revised” (ECR−RD)]. Psychother. Psychosom. Med. Psychol. 59, 215–223. 10.1055/s-2008-1067425, PMID: 18600614

[ref13] Falkenström F. Ekeblad A. Holmqvist R. (2016). Improvement of the working alliance in one treatment session predicts improvement of depressive symptoms by the next session. J. Consult. Clin. Psychol. 84, 738–751. 10.1037/ccp0000119, PMID: 27213493

[ref14] Fraley R. C. Waller N. G. Brennan K. A. (2000). An item response theory analysis of self-report measures of adult attachment. J. Pers. Soc. Psychol. 78, 350–365. 10.1037/0022-3514.78.2.350, PMID: 10707340

[ref15] Gilat I. Tobin Y. Shahar G. (2012). Responses to suicidal messages in an online support group: comparison between trained volunteers and lay individuals. Soc. Psychiatry Psychiatr. Epidemiol. 47, 1929–1935. 10.1007/s00127-012-0508-7, PMID: 22491905

[ref16] Gould M. S. Cross W. Pisani A. R. Munfakh J. L. Kleinman M. (2013). Impact of applied suicide intervention skills training on the National suicide prevention lifeline. Suicide Life Threat. Behav. 43, 676–691. 10.1111/sltb.12049, PMID: 23889494 PMC3838495

[ref17] Hatcher R. L. Gillaspy J. A. (2006). Development and validation of a revised short version of the working Alliance inventory. Psychother. Res. 16, 12–25. 10.1080/10503300500352500

[ref18] Horvath A. O. Greenberg L. S. (1989). Development and validation of the working alliance inventory. J. Couns. Psychol. 36, 223–233. 10.1037/0022-0167.36.2.223

[ref600] Hu L. T. Bentler P. M. (1999). Cutoff criteria for fit indexes in covariance structure analysis: conventional criteria versus new alternatives. Struct. Equ. Model. 6, 1–55.

[ref19] Ingram S. Ringle J. L. Hallstrom K. Schill D. E. Gohr V. M. Thompson R. W. (2008). Coping with crisis across the lifespan: the role of a telephone hotline. J. Child Fam. Stud. 17, 663–674. 10.1007/s10826-007-9180-z

[ref20] Kirchmann H. Steyer R. Mayer A. Joraschky P. Schreiber-Willnow K. Strauss B. (2012). Effects of adult inpatient group psychotherapy on attachment characteristics: an observational study comparing routine care to an untreated comparison group. Psychother. Res. 22, 95–114. 10.1080/10503307.2011.626807, PMID: 22092435

[ref400] Kirk R. E. (1996). Practical significance: a concept whose time has come. Educ. Psychol. Meas. 56, 746–759.

[ref500] Kline R. B. (2011). Principles and practice of structural equation modeling. 3rd Edn. New York: Guilford.

[ref21] Lingiardi V. Muzi L. Tanzilli A. Carone N. (2018). Do therapists’ subjective variables impact on psychodynamic psychotherapy outcomes? A systematic literature review. Clin. Psychol. Psychother. 25, 85–101. 10.1002/cpp.2131 28873269

[ref601] Little T. D. (2013). Longitudinal structural equation modeling. New York: Guilford.

[ref22] Malik S. Wells A. Wittkowski A. (2015). Emotion regulation as a mediator in the relationship between attachment and depressive symptomatology: a systematic review. J. Affect. Disord. 172, 428–444. 10.1016/j.jad.2014.10.007, PMID: 25451448

[ref23] Marmarosh C. L. Tasca G. A. (2013). Adult attachment anxiety: using group therapy to promote change. J. Clin. Psychol. 69, 1172–1182. 10.1002/jclp.22044 24151103

[ref24] Maxwell H. Tasca G. A. Ritchie K. Balfour L. Bissada H. (2014). Change in attachment insecurity is related to improved outcomes 1-year post group therapy in women with binge eating disorder. Psychotherapy 51, 57–65. 10.1037/a0031100 23398032

[ref25] Mishara B. L. Chagnon F. Daigle M. Balan B. Raymond S. Marcoux I., Berman A. (2007). Which helper behaviors and intervention styles are related to better short-term outcomes in telephone crisis intervention? Results from a silent monitoring study of calls to the U.S. 1-800-SUICIDE network. Suicide Life Threat. Behav. 37, 308–321. 10.1521/suli.2007.37.3.308, PMID: 17579543

[ref26] Mohr J. J. Gelso C. J. Hill C. E. (2005). Client and counselor trainee attachment as predictors of session evaluation and countertransference behavior in first counseling sessions. J. Couns. Psychol. 52, 298–309. 10.1037/0022-0167.52.3.298

[ref27] Morley T. E. Moran G. (2011). The origins of cognitive vulnerability in early childhood: mechanisms linking early attachment to later depression. Clin. Psychol. Rev. 31, 1071–1082. 10.1016/j.cpr.2011.06.006, PMID: 21820386

[ref28] Orlinsky D. E. Ambühl H. Rønnestad M. H. Davis J. Gerin P. Davis M., SPR Collaborative Research Network (1999). Development of psychotherapists: concepts, questions, and methods of a collaborative international study. Psychother. Res. 9, 127–153. 10.1080/10503309912331332651

[ref29] Orlinsky D. E. Rønnestad M. H. (2005). How psychotherapists develop: A study of therapeutic work and professional growth. Washington, DC, US: American Psychological Association.

[ref30] Paterson H. Völlm B. Reniers R. (2009). Personality types and mental health experiences of those who volunteer for helplines. Br. J. Guid. Couns. 37, 459–471. 10.1080/03069880903161419

[ref31] Petrowski K. Nowacki K. Pokorny D. Buchheim A. (2011). Matching the patient to the therapist: the roles of the attachment status and the helping alliance. J. Nerv. Ment. Dis. 199, 839–844. 10.1097/NMD.0b013e3182349cce, PMID: 22048135

[ref32] Rek I. Dinger U. (2015). “Training for telephone counselors from the perspective of psychotherapy research [Ausbildung zur Arbeit am telefon in der perspektive der psychotherapieforschung]” in *Telefonseelsorge interdisziplinär* (Vol. 221–238). ed. Blömeke E. H. B. (Göttingen: Vandenhoek & Ruprecht).

[ref700] Rek I. Dinger U. (2016). Who sits behind the telephone? Interpersonal characteristics of volunteer counselors in telephone emergency services. J. Couns. Psychol. 63, 429–424. 27213616 10.1037/cou0000157

[ref33] Rek I. Ehrenthal J. C. Strauss B. M. Schauenburg H. Nikendei C. Dinger U. (2018). Attachment styles and interpersonal motives of psychotherapy trainees. Psychotherapy 55, 209–215. 10.1037/pst0000154 30179028

[ref34] Romano V. Janzen J. R. I. Fitzpatrick M. R. (2009). Volunteer client attachment moderates the relationship between trainee therapist attachment and therapist interventions. Psychother. Res. 19, 666–676. 10.1080/10503300902926547, PMID: 19544186

[ref800] Rosseel Y. (2012). Lavaan: an R package for structural equation modeling and more. Version 0.5–12 (BETA). J. Stat. Softw. 48, 1–36.

[ref35] Schauenburg H. (2016). Bindung und depression. Psychother. Dialog 17, 28–31. 10.1055/s-0042-109308

[ref36] Schauenburg H. Buchheim A. Beckh K. Nolte T. Brenk K. Leichsenring F., Dinger U. (2010). The influence of psychodynamically-oriented therapists’ attachment representations on outcome and alliance in inpatient psychotherapy. Psychother. Res. 20, 193–202. 10.1080/10503300903204043, PMID: 19844844

[ref37] Smoreda Z. Licoppe C. (2000). Gender-specific use of the domestic telephone. Soc. Psychol. Q. 63, 238–252. 10.2307/2695871

[ref38] Steel C. Macdonald J. Schroder T. (2018). A systematic review of the effect of therapists’ internalized models of relationships on the quality of the therapeutic relationship. J. Clin. Psychol. 74, 5–42. 10.1002/jclp.22484, PMID: 28505384

[ref39] Taubner S. Ulrich-Manns S. Klasen J. Curth C. Möller H. Wolter S. (2014). Innere arbeitsmodelle von bindung und aversive kindheitserfahrungen bei psychotherapeuten in ausbildung. Psychother. Forum 19, 2–12. 10.1007/s00729-014-0005-4

[ref40] Taubner S. Zimmermann J. Kächele H. Möller H. Sell C. (2013). The relationship of introject affiliation and personal therapy to trainee self-efficacy: a longitudinal study among psychotherapy trainees. Psychotherapy 50, 167–177. 10.1037/a0029819 23066923

[ref41] Taylor P. Rietzschel J. Danquah A. Berry K. (2015). Changes in attachment representations during psychological therapy. Psychother. Res. 25, 222–238. 10.1080/10503307.2014.886791 24559454

[ref42] TelefonSeelsorge (2016). *Gesamtstatistik für das Jahr 2016* Available at: http://www.telefonseelsorge.de/?q=node/42# (Accessed February 10, 2017).

[ref43] Wilmers F. Munder T. Leonhart R. Herzog T. Plassmann R. Barth J. (2008). Die deutschsprachige version des working alliance inventory - short revised (WAI-SR) - Ein schulenübergreifendes, ökonomisches und empirisch validiertes Instrument zur Erfassung der therapeutischen Allianz. Klin. Diagn. Eval. 1, 343–358.

